# Effect of the Molecular Mass of Tremella Polysaccharides on Accelerated Recovery from Cyclophosphamide-Induced Leucopenia in Rats

**DOI:** 10.3390/molecules17043609

**Published:** 2012-03-23

**Authors:** Rui-Zhi Jiang, Ying Wang, Hao-Ming Luo, Yan-Qiu Cheng, Ying-Hong Chen, Yang Gao, Qi-Pin Gao

**Affiliations:** 1 Jilin Academy of Chinese Medicine and Material Medica Science, Changchun 130012, Jilin, China; Email: zhongyanjrz@yahoo.com.cn (R.-Z.J.); 2 College of Pharmacy, Jilin University, Changchun 130021, Jilin, China; Email: Luo.haoming@163.com; 3 Center for New Medicine Research, Changchun University of Chinese Medicine, Changchun 130021, Jilin, China

**Keywords:** tremella polysaccharide, hydrolysis, molecular mass, cyclophosphamide-induced leucopenia

## Abstract

The body of tremella were decocted with water, and hydrolyzed with 0.1 mol/L hydrochloric acid for different times, giving tremella polysaccharides with six molecular mass values. The structures of all the tremella polysaccharides had non-reducing terminals of β-D-pyranglucuronide, the backbone was composed of (1→3)-linked β-D-manno-pyranoside, and the side chain composed of (1→6)-linked β-D-xylopyranoside was attached to the C_2_ of the backbone mannopyranoside. Immunomodulatory effect studies indicated that tremella polysaccharides increased the counts of leukocytes in the peripheral blood which were significantly lowered by cyclophosphamide, and the lower the molecular mass of the tremella polysaccharide, the better this effect was.

## 1. Introduction

Cancer is one of the leading causes of death in the world. Chemotherapeutics are often used to inhibit the proliferation of cancer cells. Cyclophosphamide (Cy) is the most widely used alkylating agent in cancer chemotherapy to date. The anti-tumor effect of Cy is proportional to the dose of Cy administered, often resulting in unwanted immunosuppressive and cytotoxic effects [1]. Chemotherapy-induced leukopenia leads to significant morbidity and mortality, a major limiting factor in clinical chemotherapy without efficacious remedies. In Traditional Chinese Medicine, co-administration of immunomodulatory agents and chemotherapy drugs is typically used to improve the immunity potential. In many oriental countries, several immunoceuticals composed of polysaccharides are widely used, such as lentinan (LT), schizophyllan and krestin. Those immunomodulatory agents often act by inducing lymphocyte proliferation and cytokine production, and they have protective effects toward the hematopoietic function of bone marrow and immune organs [2].

The body of tremella (*Tremella fuciformis*) is a popular food and herbal medicine, widely used in Asian countries as a tonic [3]. Tremella polysaccharide (TP) has received extensive attention. TP consist of a (1→3)-mannan backbone with small xylose-, glucose-, arabinose-, fucose- and glucuronic acid-containing side chains [4]. It was reported that TP exerted an anti-aging effect by increasing the levels of superoxide dismutase (SOD), a key antioxidant enzyme in brain and liver cells [5], and other pharmacological activities, including cytokine-stimulating, anti-tumor, anti-diabetic, antiinflammatory, vascular-stimulating, cholesterol-lowering, antiallergic and hepatoprotective effects have been reported [6].

In this paper, we investigated the structural characterisation of TPs with six different molecular mass values, and their immunomodulatory effect against cyclophosphamide-induced leucopenia in rats. We found that TP showed the immunomodulatory effects against cyclophosphamide-induced leucopenia in rats in a dose-dependent manner, and the lower the molecular mass of TP was, the better was the activity. 

## 2. Results and Discussion

**Figure 1:**
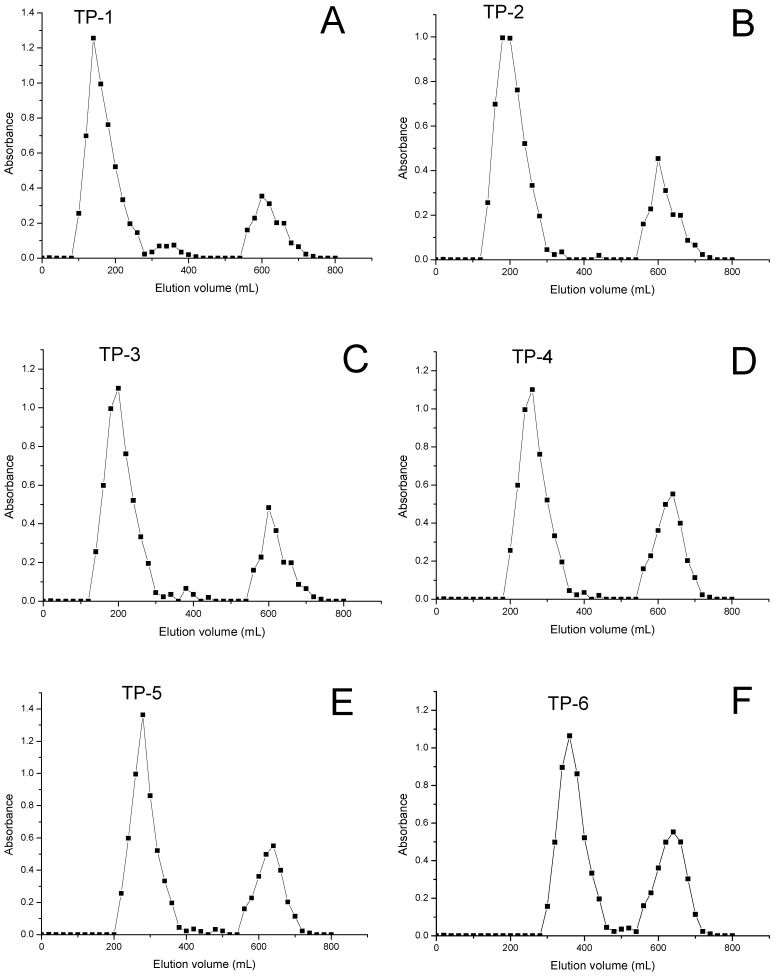
Elution profile by Sephadex G-150. Absorbance at 490 nm from the phenol-sulfuric acid assay. Column void volumes were 0-50 mL and 550 mL-600 mL. **A**: elution profile of TP-1; **B**: elution profile of TP-2; **C**: elution profile of TP-3; **D**: elution profile of TP-4; **E**: elution profile of TP-5; **F**: elution profile of TP-6.

The aqueous extract (TP) of the body of tremella was hydrolyzed with 0.1 mol/L hydrochloric acid at 90 °C for 0.5 h, 1.5 h, 2 h, 2.5 h, 3 h and 3.5 h. The six hydrolysates were separately applied onto a Sephadex G-150 column, and six samples (named TP-1, TP-2, TP-3, TP-4, TP-5 and TP-6) were thus obtained (Figure 1).

All the HPLC profiles of TP-1, TP-2, TP-3, TP-4, TP-5 and TP-6 showed single peaks, so TP-1 to TP-6 were homogeneous materials. The average molecular masses of TP-1, TP-2, TP-3, TP-4, TP-5 and TP-6 were 500,000 Da, 200,000 Da, 100,000 Da, 30,000 Da, 10,000 Da and 4,000 Da, respectively, calculated using GPC software.

Compared with TP-1, TP-2, TP-3, TP-4 and TP-5, the total carbohydrates content of TP-6, which was 83.1%, was the highest, (Table 1). The monosaccharide components of TP-1, TP-2, TP-3, TP-4, TP-5 and TP-6 as analyzed by HPLC were mannose, xylose and glucuronic acid, and the content of mannose was the highest (Table 1). The above results implied that TP-1 to TP-6 were homogeneous tremella polysaccharides.

**Table 1:** Physicochemical properties, molecular mass and monosaccharide compositions of TP, TP-1, TP-2, TP-3, TP-4, TP-5 and TP-6.

	TP	TP-1	TP-2	TP-3	TP-4	TP-5	TP-6
Yield (%)	26.33	65.80	60.82	59.37	61.25	63.64	61.29
Molecular mass (Da)	1,500,000	500,000	200,000	100,000	30,000	10,000	4,000
Carbohydrate (%) ^a^	80.25	76.50	78.20	79.07	81.36	80.84	83.10
Uronic acid (%) ^a^	11.16	19.80	21.10	20.33	19.09	20.27	19.50
Protein (%) ^a^	tr ^b^	tr ^b^	tr ^b^	tr ^b^	tr ^b^	tr ^b^	tr ^b^
Monosaccharide composition (mol%) ^c^							
Mannose	57.50	60.45	52.65	54.46	58.82	60.02	62.67
xylose	32.09	18.91	20.66	20.14	19.36	18.11	17.31
Glucuronic acid	10.14	20.64	21.44	25.40	21.82	21.87	19.77

^a^ Calculated as weight percent of applied material; ^b^ Trace; ^c^ Mole percent of total carbohydrate content.

The FT-IR spectra of TP-1, TP-2, TP-3, TP-4, TP-5 and TP-6 (data not shown) showed presence of bands typical for those of saccharides and the the band at 896 cm^−1^ indicated the presence of β-linked sugar residues [7].

**Figure 2:**
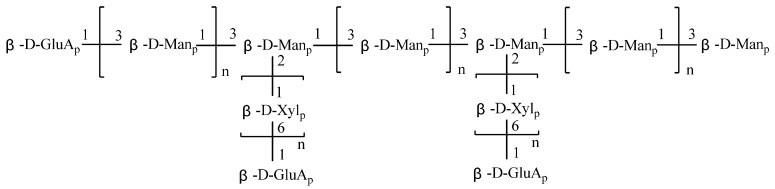
The structure of TP-1 to TP-6 (n ≥ 1).

As shown in Table 2, glucuronic acid was reduced to be glucose before methylation, so 2,3,4,6-Me_4_-D-Glc is the methylation product of glucuronic acid. The structures of TP-1, TP-2, TP-3, TP-4, TP-5 and TP-6 had non-reducing terminals of β-D-pyranglucuronide, the backbone was composed of (1→3)-linked β-D-mannopyranoside, and the side chain, composed of (1→6)-linked β-D-xylopyranoside, was attached through C_2_ of the backbone mannosyl residues. The structure of TP-1 to TP-6 (shown in Figure 2) is consistent with tremella polysaccharide, which means the tremella polysaccharide has repeating units which can be prepared by acid hydrolysis. Because the glycosidic bond connected with glucuronic acid was hard to hydrolyze by acid hydrolysis [8], the side chains of TP contained glucuronic acid that was not hydrolyzed. 

**Table 2:** Methylation analysis of TP-1, TP-2, TP-3, TP-4, TP-5 and TP-6.

Retention time (min)	Product	TP (mol%) ^a^	TP-1 (mol%) ^a^	TP-2 (mol%) ^a^	TP-3 (mol%) ^a^	TP-4 (mol%) ^a^	TP-5 (mol%) ^a^	TP-6 (mol%) ^a^
14.348	2,3,4-Me_3_-D-Xyl	15.21	18.02	20.18	19.91	18.62	20.51	18.05
16.550	2,3-Me_2_-D-Xyl	3.19	－	－	－	－	－	－
17.567	2,3,4,6-Me_4_-D-Man	9.18	－	－	－	－	－	－
19.669	2,4,6-Me_3_-D-Man	32.19	30.73	34.68	33.26	36.19	33.37	35.59
20.956	4,6-Me_2_-D-Man	21.01	31.14	22.79	27.45	24.41	21.84	25.74
17.909	2,3,4,6-Me_4_-D-Glc	18.66	20.11	22.35	19.38	20.78	24.28	20.62

^a^ Mole percent of total carbohydrate content.

The TP didn’t showed immunomodulatory effects against cyclophosphamide-induced leucopenia in rats by intraperitoneal injection (data not shown). This is because the molecular mass of TP was high, the viscosity was too high to dissolve it properly. As shown in Table 3, the administration of TP-1 to TP-6, respectively, once a day increased the counts of leukocytes in the peripaeral blood which were significantly lowered by Cy in a dose-dependent manner. TP-6 showed the best immunomodulatory effect against cyclophosphamide-induced leucopenia in rats (in Table 3), the results of both low dose and high dose group were better than matrine group from day 3 to day 9. By day 9, the leukocyte numbers of TP-6 group with 200 mg/kg/d had returned to the level of normal rats.

**Table 3:** Accelerated recovery from cyclophosphamide-induced leucopenia in rats (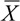 ± SD, n = 10).

Group	Leukocyte numbers (109/L)				
	0 d	3 d	6 d	9 d	12 d
Normal group	8.21 ± 1.29	8.15 ± 1.57	8.19 ± 1.36	7.95 ± 0.90	8.04 ± 1.10
Model group	8.30 ± 1.11	4.26 ± 0.78	3.93 ± 0.67	5.03 ± 0.80	6.38 ± 0.77
Matrine group (100 mg/kg/d)	8.25 ± 1.22 *	5.86 ± 1.23 *	5.88 ± 1.06*	6.02 ± 1.24 *	7.85 ± 1.37 *
TP-1 (100 mg/kg/d)	8.19 ± 1.21	4.48 ± 1.09	4.43 ± 1.41	4.72 ± 0.89	5.02 ± 0.99
TP-1 (200 mg/kg/d)	8.23 ± 1.11	4.53 ± 1.32	4.39 ± 1.09	4.96 ± 1.37	5.32 ± 1.24
TP-2 (100 mg/kg/d)	8.27 ± 1.22	4.83 ± 1.21	4.46 ± 1.19	5.70 ± 1.15	6.78 ± 0.61
TP-2 (200 mg/kg/d)	8.07 ± 1.33	4.78 ± 1.44	4.36 ± 0.74	5.94 ± 1.21	6.88 ± 1.16
TP-3 (100 mg/kg/d)	8.31 ± 1.23	5.65 ± 1.47	4.97 ± 1.09	5.84 ± 0.92	7.12 ± 0.93
TP-3 (200 mg/kg/d)	8.12 ± 1.11	5.71 ± 1.32	5.01 ± 0.83	5.95 ± 0.99	7.46 ± 1.19
TP-4 (100 mg/kg/d)	8.19 ± 0.89	5.52 ± 1.38	5.05 ± 1.65	5.79 ± 1.07	7.09 ± 1.24
TP-4 (200 mg/kg/d)	8.11 ± 1.01	5.69 ± 1.11	5.26 ± 1.41	6.03 ± 0.98	7.13 ± 1.18
TP-5 (100 mg/kg/d)	8.25 ± 1.11	5.83 ± 1.13	6.03 ± 0.99	6.47 ± 1.25	6.74 ± 1.12
TP-5 (200 mg/kg/d)	8.19 ± 1.13	5.91 ± 1.28	6.33 ± 1.15	7.29 ± 1.08	7.11 ± 1.32
TP-6 (100 mg/kg/d)	8.20 ± 1.21	5.90 ± 1.27	6.51 ± 1.11	6.84 ± 1.09	6.69 ± 0.90
TP-6 (200 mg/kg/d)	8.13 ± 1.27	5.97 ± 1.14 *	6.59 ± 1.18 *	7.80 ± 1.02 *	7.02 ± 1.13 *

* *p* < 0.05, compared with cyclophosphamide-induced leucopenia group.

Cy reduces the bone marrow cellularity and the number of leucocytes in peripheral blood and the thymidine incorporation in bone marrow cells [9]. TP-6 could increase the counts of leukocytes in the peripheral blood which were significantly lowered by Cy, probably because it could enhance the haemopoiesis of bone marrow. The results suggested that TP-6 could increase the counts of bone marrow cellularity and the DNA in bone marrow cells.

Previously, we found that after oral administration of TP only trace amounts (about 0.4%) was passed through the gastrointestinal tract into the blood and absorbed by organs [10]. Among TP-1, TP-2, TP-3, TP-4, TP-5 and TP-6, the results of the physicochemical properties, monosaccharide compositions and structure analysis are similar, but the molecular mass is different. From the results in Table 3, we inferred that the lower the molecular mass of TP, the activity was better. Perhaps this is because the permeability is proportional to the molecular mass, so low molecular mass TP could easily pass through cell membranes, be absorbed by organs and show the effects.

## 3. Experimental

### 3.1. General

Total carbohydrates, uronic acid and protein content deteminations were performed by the phenol-sulfuric acid [11], carbazole [12] and Bradford methods [13], respectively, using D-mannose (≥99%, Sigma Chemical Co., Santa Clara, CA, USA), D-glucuronic acid (≥99%, Sigma Chemical Co.) and bovine serum albumin (≥99%, Sigma Chemical Co.) as standards, respectively. A HPLC instrument (Shimadzu, city Japan) equipped with a refractive index detector (Shimadzu) on an OH-pak column (SB-802, 802.5, 803, 804, Shodex, Tokyo, Japan) and with GPC software (Saiertai Technology Co., Ltd., Hangzhou, China) were used to obtain the purity and molecular mass of each sample. For HPLC, the monosaccharide compositions of TP were analyzed as PMP derivatisation [14,15].

### 3.2. The Preparation of Six Kinds of Molecular Mass Tremella Polysaccharide

Body of tremella was purchased from a local market in Changchun City (China). Tremella (1 Kg) was cut into small pieces and decocted three times with water (50 L), then the aqueous solutions were combined into one portion, and evaporated. The aqueous extract was hydrolyzed with 0.1 mol/L hydrochloric acid at 90 °C for 0.5 h, 1.5 h, 2 h, 2.5 h, 3 h and 3.5 h to obtained six hydrolysates. After hydrolysis, 20% NaOH was added to the six hydrolysates to adjuated the pH to 7.0, then they were centrifuged at 3000 rpm for 10 min, and the supernatants forced through a Cellulose Superfiltration System to give the high molecular weight fractions, which were then lyophilised. The six dry samples were separately applied into a Sephadex G-150 column (2.5 × 90 cm, from Sigma Chemical Co.), and eluted with water (800 mL). Major fractions were collected according to their elution patterns. Six samples (named TP-1, TP-2, TP-3, TP-4, TP-5 and TP-6) were obtained (Figure 1).

### 3.3. IR Study

The FT-IR spectrum was acquired using a Bruker (Germany) Vertex 70 FTIR instrument. The sample was pressed into KBr pellets and the spectra were recorded in a transmittance mode over a wavelength range between 4,000 and 400 cm^−1^.

### 3.4. Methylation Analysis

Samples were methylated once by the Ciucanu method [16]. The resulting partially methylated alditol acetates were analysed by GC-MS. GC-MS was performed on a Trace-MS instrument (Finnigan, Santa clara, CA, USA) using a DB-17HT capillary colum (30 m × 0.25 mm, J&W Scientific, Folsom, CA, USA). The injection temperature was 200 °C. The column temperature was kept at 50 °C for 2 min after sample injection, increased to 150 °C at 50 °C/min, kept at 150 °C for 1 min, then increased to 250 °C at 4 °C/min. The mass spectra were recorded in the positive ion electron ionization mode.

### 3.5. Accelerated Recover from Cyclophosphamide-Induced Leucopena in Rats

Young male (75) and young female (75) Kunming rats (weight 19–21 g) were supplied by the School of Pharmacy, Jilin University. Animals were randomly divided into 14 experimental groups (10 per group): normal group, model group, matrine group (matrine injection, 100 mg/kg/d), TP-1 group (100, 200 mg/kg/d), TP-2 group (100, 200 mg/kg/d), TP-3 group (100, 200 mg/kg/d), TP-4 group (100, 200 mg/kg/d), TP-5 group (100, 200 mg/kg/d) and TP-6 group (100, 200 mg/kg/d). Matrine injection, which was a commercial drug from China was used as positive drug, and TP-1 to TP-6 used as sample drugs. Rats were housed under specific pathogen-free conditions.

Rats were intraperitoneally injected with 100 mg/kg/d Cy from day 1 to day 3, except the normal group. From day 4 to day 12, the rats of the normal, model and sample groups were orally administered the following: Normal group, normal saline; model group, normal saline; sample groups, 100 or 200 mg/kg/d body weight TP, the rats of the matrine group received the drug by tail vein injection.

Aliquots (0.02 mL) of peripheral blood were collected from the tail vein by making a small cut, diluted with 0.08 mL of azide-free balanced electrolyte solution, Isoton® II (Coulter Scientific Japan, Tokyo, Japan), and mixed with 0.1 mL of Türk solution (Wako, Osaka, Japan). Leukocyte numbers were counted microscopically by a hemocytometer [17]. The data was analyzed by ANOVA. Values of p < 0.05 were considered statistically significant.

## 4. Conclusions

Six kinds of molecular mass tremella polysaccharides (TP-1, TP-2, TP-3, TP-4, TP-5 and TP-6) were obtained by hydrolyzing TP. The average molecular mass of TP-1, TP-2, TP-3, TP-4, TP-5 and TP-6 were 500,000 Da, 200,000 Da, 30,000 Da, 100,000 Da, 10,000 Da and 4,000 Da, respectively. The physicochemical properties, monosaccharide compositions and structure analysis show that TP-1, TP-2, TP-3, TP-4, TP-5 and TP-6 are similar and they possess repeating unit structures.

Immunomodulatory effect studies showed that TP increased the counts of leukocytes in the peripheral blood, which were significantly lowered by Cy in a dose-dependent manner. The lower the molecular mass of TP was, the better the activity was, so TP-6, which had the lowest molecular mass, showed the best activity. In the next study, we plan to find the optimum molecular mass of TP showing the optimum immunomodulatory effects against cyclophosphamide-induced leucopenia in rats.
